# Association of the Haptoglobin Gene Polymorphism With Cognitive Function and Decline in Elderly African American Adults With Type 2 Diabetes: Findings From the Action to Control Cardiovascular Risk in Diabetes–Memory in Diabetes (ACCORD-MIND) Study

**DOI:** 10.1001/jamanetworkopen.2018.4458

**Published:** 2018-11-09

**Authors:** Michal S. Beeri, Hung-Mo Lin, Mary Sano, Ramit Ravona-Springer, Xiaoyu Liu, Barbara B. Bendlin, Carey E. Gleason, Elizabeth Guerrero-Berroa, Laili Soleimani, Lenore J. Launer, Scott Ehrenberg, Orit Lache, Yaakov K. Seligman, Andrew P. Levy

**Affiliations:** 1 Department of Psychiatry, Icahn School of Medicine at Mount Sinai, New York, New York; 2 The Joseph Sagol Neuroscience Center, Sheba Medical Center, Ramat Gan, Israel; 3 Department of Environmental Medicine and Public Health, Icahn School of Medicine at Mount Sinai, New York, New York; 4 The Memory and Psychogeriatric Clinic, Sheba Medical Center, Tel Hashomer, Israel; 5 Division of Geriatrics and Gerontology, Department of Medicine, School of Medicine and Public Health, University of Wisconsin–Madison; 6 Department of Psychology, Lehman College, The City University of New York, Bronx; 7 Laboratory of Epidemiology and Population Science, National Institute on Aging, National Institutes of Health, Bethesda, Maryland; 8 currently a student at Rappaport Faculty of Medicine, Technion-Israel Institute of Technology, Haifa, Israel; 9 Department of Medicine, Rappaport Faculty of Medicine, Technion-Israel Institute of Technology, Haifa, Israel

## Abstract

**Importance:**

African American individuals have higher dementia risk than individuals of white race/ethnicity. They also have higher rates of type 2 diabetes, which may contribute to this elevated risk. This study examined the association of the following 2 classes of alleles at the haptoglobin (Hp) locus that are associated with poor cognition, cardiovascular disease, and mortality: Hp 1-1 (associated with poor cognition and cerebrovascular disease) and Hp 2-1 and Hp 2-2 (associated with greater risk of myocardial infarction and mortality). An additional polymorphism in the promoter region of the Hp 2 allele, restricted to individuals of African descent, yields a fourth genotype, Hp 2-1m. African American adults have a higher prevalence of Hp 1-1 (approximately 30%) compared with individuals of white race/ethnicity (approximately 14%), but the potential role of the Hp genotype in cognition among elderly African American individuals with type 2 diabetes is unknown.

**Objective:**

To assess the association of the Hp genotypes with cognitive function and decline in elderly African American adults with type 2 diabetes.

**Design, Setting, and Participants:**

This cohort study used publicly available data and specimens from the Action to Control Cardiovascular Risk in Diabetes–Memory in Diabetes (ACCORD-MIND) study to investigate the association of the Hp genotypes with cognitive function and decline in 466 elderly African American participants with type 2 diabetes. The hypothesis was that the Hp 1-1 genotype compared with the other genotypes would be associated with more cognitive impairment and faster cognitive decline in elderly African American adults with type 2 diabetes. The initial ACCORD trial was performed from October 28, 1999, to September 15, 2014. This was a multicenter clinical study performed in an academic setting.

**Exposures:**

The Hp genotypes were determined from serum samples by polyacrylamide gel electrophoresis and by enzyme-linked immunosorbent assay.

**Main Outcomes and Measures:**

The Mini-Mental State Examination (MMSE) was used to measure cognitive function and change after 40 months. The MMSE score ranges from 0 to 30 points; higher scores represent better cognition. Associations were examined with analysis of covariance and linear regression, adjusting for age, sex, education, baseline glycated hemoglobin level, systolic blood pressure, diastolic blood pressure, cholesterol level, creatinine level, and treatment arm (intensive vs standard). The cognitive change model adjusted also for the baseline MMSE score.

**Results:**

Among 466 African American study participants (mean [SD] age, 62.3 [5.7] years), 64.8% were women, and the genotype prevalences were 29.4% (n = 137) for Hp 1-1, 36.1% (n = 168) for Hp 2-1, 10.9% (n = 51) for Hp 2-1m, and 23.6% (n = 110) for Hp 2-2. The groups differed in their baseline MMSE scores (*P* = .006): Hp 1-1 had the lowest MMSE score (mean [SE], 25.68 [0.23]), and Hp 2-1m had the highest MMSE score (mean [SE], 27.15 [0.36]). Using the least squares method, the 40-month decline was significant for Hp 1-1 (mean [SE], −0.41 [0.19]; *P* = .04) and for Hp 2-2 (mean [SE], −0.68 [0.21]; *P* = .001). However, the overall comparison across the 4 groups did not reach statistical significance for the fully adjusted model. The interaction of age with the Hp 1-1 genotype on MMSE score decline estimate per year change was significant (mean [SE], −0.87 [0.37]; *P* = .005), whereas it was not significant for Hp 2-1 (mean [SE], 0.06 [0.37]; *P* = .85), Hp 2-1m (mean [SE], −0.06 [0.51]; *P* = .89), and Hp 2-2 (mean [SE], −0.44 [0.41]; *P* = .29), indicating that cognitive decline in Hp 1-1 carriers was accentuated in older ages, whereas it was not significant for the other Hp genotypes.

**Conclusions and Relevance:**

In this study, the Hp 1-1 genotype, which is 2-fold (approximately 30%) more prevalent among African American individuals than among individuals of white race/ethnicity, was associated with poorer cognitive function and greater cognitive decline than the other Hp genotypes. The Hp gene polymorphism may explain the elevated dementia risk in African American adults. The neuropathological substrates and mechanisms for these associations merit further investigation.

## Key Points

**Question:**

Is the haptoglobin genotype associated with cognition in African American adults with type 2 diabetes?

**Findings:**

In this cohort study of 466 African American adults with type 2 diabetes, haptoglobin 1-1 carriers had the poorest cognitive function, while haptoglobin 2-1m carriers had the highest cognitive function. Older haptoglobin 1-1 carriers had greater cognitive decline over time compared with the other genotypes.

**Meaning:**

The haptoglobin gene polymorphism may contribute to cognitive impairment in African American adults who have type 2 diabetes.

## Teaser

This cohort study assesses the association of haptoglobin genotypes with cognitive function and decline in elderly African American adults with type 2 diabetes.

## Introduction

African American adults have a significantly higher risk of developing dementia and Alzheimer disease than other racial/ethnic groups,^1,2,3^ even after adjusting for age, sex, and education.^1^ In addition to nongenetic factors, such as poor nutrition^4^ and obesity,^5^ higher rates of cerebrovascular disease and cardiovascular risk factors^6^ (especially type 2 diabetes^7^) in African American adults suggest that the genetics of vascular disease may underlie this increased risk of dementia.

Haptoglobin (Hp) is a hemoglobin-binding protein synthesized in the liver and the brain. Two classes of alleles at the Hp locus yield 3 genotypes, Hp 1-1, Hp 2-1, and Hp 2-2. An additional polymorphism in the promoter region of the Hp 2 allele, restricted to individuals of African descent, yields a fourth genotype, Hp 2-1m.^8,9^ The biophysical properties of the Hp polymers formed in these 4 Hp genotypes are distinct, but little is known about the Hp 2-1m biology. The associations of Hp with health—studied almost solely in individuals of white race/ethnicity—are primarily in patients with type 2 diabetes.^10^ Hp 2-2 is associated with increased risk of myocardial infarction and mortality.^11,12^ Hp 1-1 has been linked to cerebral small vessel disease (eg, lacunar infarcts and white matter lesions).^13,14,15,16^ Consistent with the latter, it has been shown in a cohort of elderly white patients with type 2 diabetes that Hp 1-1 carriers have poorer cognitive function compared with Hp 2-1 and Hp 2-2 carriers.^17^ A study^18^ examining the prevalence of the Hp genotypes across different populations reported that African American adults have a higher prevalence of Hp 1-1 (approximately 30%) compared with individuals of white race/ethnicity (approximately 14%), but the potential role of the Hp genotype in poorer cognition and cognitive decline in elderly African American adults with type 2 diabetes has not been studied.

We took advantage of publicly available data and specimens from the Action to Control Cardiovascular Risk in Diabetes–Memory in Diabetes (ACCORD-MIND) study to investigate the association of the Hp genotypes with cognitive function and decline in 466 elderly African American participants. We hypothesized that the Hp 1-1 genotype compared with the other genotypes would be associated with more cognitive impairment and faster cognitive decline in elderly African American adults with type 2 diabetes.

## Methods

### ACCORD Study Design

The rationale, design, eligibility criteria, and glycemia intervention of the initial ACCORD trial have been reported previously.^19^ The ACCORD trial was performed from October 28, 1999, to September 15, 2014. Briefly, this was a multicenter clinical study in an academic setting that recruited volunteers with type 2 diabetes and a glycated hemoglobin level of 7.5% or more who were between the ages of 40 and 79 years and had cardiovascular disease. All 10 251 patients were randomly assigned to receive comprehensive intensive therapy targeting a glycated hemoglobin level of less than 6.0% or to receive standard therapy targeting a glycated hemoglobin level of 7.0% to 7.9%. The study protocol was approved by the institutional review boards of its 77 participating sites, as well as a review panel at the National Heart, Lung, and Blood Institute. All patients provided written informed consent. This study followed Strengthening the Reporting of Observational Studies in Epidemiology (STROBE) reporting guideline for cohort studies.

As a substudy, the ACCORD-MIND study^20^ aimed to examine the association of intensive vs standard glycemic control with cognitive function and decline. In total, 2977 ACCORD trial participants were included in this substudy, 466 of whom were African American individuals who had complete baseline information on the Mini-Mental State Examination (MMSE) score and relevant covariates. Cognition was measured at baseline and at the 20-month and 40-month visits.

### Hp Genotyping

**Figure 1.**
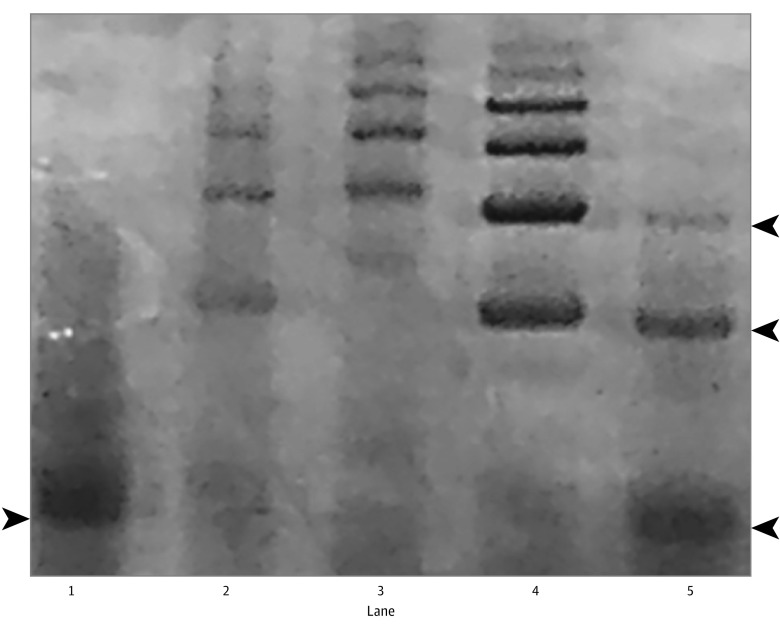
Determination of Haptoglobin (Hp) Genotype by Nondenaturing Polyacrylamide Gel Electrophoresis Demonstration of the 4 possible Hp genotypes by polyacrylamide gel electrophoresis. Each of the 4 Hp genotypes has a signature banding pattern resulting from differences in stoichiometry of the polymeric composition of the different Hp genotypes.^13^ In lane 1, Hp 1-1 has a single band corresponding to the Hp 1-1 dimer (see the arrowhead pointing to the right). In lane 5, Hp 2-1m has a band present in Hp 1-1 and 2 additional bands corresponding to Hp 2-1 polymers (trimer and quatermer [see the arrowheads pointing to the left]). In lanes 2 and 4, Hp 2-1 has the bands present in Hp 2-1, as well as higher-order Hp 2-1 polymers. In lane 3, Hp 2-2 has only Hp 2-2 trimers or higher-order Hp 2-2 polymers.

Through a request for data and serum samples to the Biologic Specimen and Data Repository Information Coordinating Center (BIOLINCC) system of the National Heart, Lung, and Blood Institute,^21^ specimens were sent to the Technion–Israel Institute of Technology (Haifa) and were genotyped for Hp by polyacrylamide gel electrophoresis (PAGE)^22^ and by enzyme-linked immunosorbent assay (ELISA),^23^ with no knowledge of any participant-related information. The ELISA assay provides a spectrophotometric readout that has been validated as having greater than 99% accuracy for Hp 1-1, Hp 2-1, and Hp 2-2.^23,24^ However, to our knowledge, the present study is first in using the ELISA assay for assessing Hp 2-1m, whose reading was (as expected) intermediate between Hp 1-1 and Hp 2-1. The PAGE method uses hemoglobin enrichment of serum^22^ and provides a signature banding pattern for Hp 1-1, Hp 2-1, Hp 2-1m, and Hp 2-2 (Figure 1). Hp 2-1m is found only in African American individuals and notably was not seen in any of the samples analyzed from individuals of white race/ethnicity (eg, the Israel Diabetes and Cognitive Decline study^25^), consistent with previous studies among white individuals^26^ and nonwhite individuals,^27,28^ and testifies to the accuracy of the Hp typing.^23,24^

### Hp Protein Quantitation

To investigate the potential role of Hp protein levels on cognition and in the association of the Hp genotype with cognition, we quantified Hp protein levels. A 96-well microtiter dish (96-Well Microtiter Microplates; Nunc) was coated with 100 µL of rabbit anti-Hp in phosphate-buffered saline (PBS) (antibody titer 1:3000; Dako) and incubated overnight. The wells were rinsed with wash buffer (PBS with 0.05% TWEEN 20) and then incubated with blocking buffer (PBS with 5% bovine serum albumin and 0.05% TWEEN 20) for 2 hours at room temperature. The wells were then rinsed with wash buffer; serum samples diluted 1:100 000 or Hp standards (0.1-1.0 ng) in blocking buffer were added, and the plate was incubated overnight. The next day, the wells were washed, and mouse anti-Hp (1:10 000; Sigma) was added in blocking buffer for 1 hour. Thereafter, the wells were washed, and horseradish peroxidase anti-mouse antibody (1:20 000; Jackson ImmunoResearch Laboratories) in blocking buffer was added for 30 minutes. The wells were washed again, and substrate was added for 20 minutes, followed by stop solution (1N sulfuric acid). The plate was read at 450 nm in an ELISA reader.

### Hp Standards

To evaluate the role of Hp protein levels in cognition and in the association of the Hp genotypes with cognition, Hp protein was obtained by immunoaffinity purification on an anti-Hp column. The Hp protein levels of the standards were determined using the following extinction coefficients (for solutions of 1 mg/mL), in ascending order: 1.18 for Hp 2-2, 1.21 for Hp 2-1, 1.23 for Hp 2-1m, and 1.24 for Hp 1-1.

### Mini-Mental State Examination

The MMSE^29^ is a global cognitive screening instrument that assesses orientation, memory, attention/concentration, language, and visual construction. The MMSE score ranges from 0 to 30 points; higher scores represent better cognition.

### Statistical Analysis

Descriptive data are reported as number (percentage) or mean (SD), as appropriate. Unadjusted bivariate analyses included 1-way analysis of variance or χ^2^ test, as appropriate, to compare Hp genotype differences relative to sociodemographic and clinical variables. The sociodemographic variables included baseline age, sex, and education (high school or less, some college or technical school, high school graduate or general equivalency diploma, and college graduate or more). The clinical variables included baseline glycated hemoglobin level, systolic blood pressure, diastolic blood pressure, cholesterol level, creatinine level, and cardiovascular disease (which includes stroke and myocardial infarction). An indicator variable for treatment arm (intensive vs standard) was also included.

The primary analysis used the MMSE to assess the association of the Hp genotypes with MMSE score and measure cognitive function and change after 40 months. eTable 1 in the Supplement lists the distribution of the MMSE scores in the 4 genotypes. Two sets of linear regression models were fit. The primary model adjusted for baseline age, sex, and education. In a second model, we further adjusted for baseline clinical factors (glycated hemoglobin level, systolic blood pressure, diastolic blood pressure, cholesterol level, creatinine level, and cardiovascular disease) and the glycemic control study arm.

The association between Hp genotypes and MMSE score was examined both cross-sectionally and longitudinally using the same primary and secondary sets of adjustment models as described in the previous paragraph. The cross-sectional analyses compared the MMSE score differences measured at baseline across the 4 Hp genotypes, while the longitudinal analysis using the mixed model with random participants focused on the change in MMSE score from baseline to the 20-month and 40-month follow-ups, with additional adjustment for the baseline MMSE score and use of blood pressure and oral glycemic control medications at the time of follow-up visits. For the change models, we assumed that missing data and dropouts were at random (ie, the unobserved data could be anticipated using the covariates considered in the model through the expectation-maximization algorithm).

The ACCORD-MIND study participants were young (mean age, 62 years) in the context of a study on cognitive decline, while the MMSE shows greater variability in older individuals.^30^ Therefore, we performed secondary analyses to examine the interaction of age with Hp genotypes on the MMSE score change. We hypothesized that, at older ages, there would be greater cognitive decline in those with the Hp 1-1 genotype. To investigate this, we first plotted the MMSE score change against the baseline age by genotype using nonparametric local linear regression (locally weighted smoothing 80% smoothed with 95% CI). We then repeated the aforementioned longitudinal models (in the previous paragraph) but allowed each genotype to have its specific age association (ie, replacing the baseline age variable with the interaction term of baseline age with each individual genotype). Statistical analysis was performed using a software program (SAS, version 9.4; SAS Institute Inc). Two-sided *P* < .05 was defined as the significance level in all statistical tests.

## Results

Table 1 lists the sociodemographic and clinical characteristics of the participants by Hp genotype. Among 466 African American study participants (mean [SD] age, 62.3 [5.7] years), 64.8% were women, and the genotype prevalences were 29.4% (n = 137) for Hp 1-1, 36.1% (n = 168) for Hp 2-1, 10.9% (n = 51) for Hp 2-1m, and 23.6% (n = 110) for Hp 2-2. The Hp groups differed in baseline glycated hemoglobin levels, with the highest level for the Hp 2-2 genotype (8.8%) and the lowest level for the Hp 2-1m genotype (8.2%). Otherwise, there were no significant differences among the 4 groups.

**Table 1.** Baseline Characteristics of the Sample and by the Hp Genotype

Variable	Overall	Hp 1-1	Hp 2-1	Hp 2-1m	Hp 2-2	*P* Value
Baseline, No. (%)^a^	466 (100)	137 (29.4)	168 (36.1)	51 (10.9)	110 (23.6)	NA
40-mo Follow-up, No. (%)^a^	409 (100)	120 (29.3)	146 (35.7)	46 (11.2)	97 (23.7)	NA
Age, mean (SD), y	62.3 (5.7)	61.7 (5.6)	62.6 (5.7)	63.7 (6.4)	61.8 (5.3)	.10
Female, No. (%)^b^	302 (64.8)	94 (68.6)	113 (67.3)	33 (64.7)	62 (56.4)	.19
High school or less, No. (%)^b^	236 (50.6)	62 (45.3)	93 (55.4)	29 (56.9)	52 (47.3)	.22
Glycated hemoglobin level, mean (SD), %	8.6 (1.2)	8.6 (1.2)	8.5 (1.1)	8.2 (0.9)	8.8 (1.2)	.007
SBP, mean (SD), mm Hg	139.2 (18.5)	138.8 (18.1)	138.2 (18.5)	142.3 (18.1)	139.9 (19.3)	.54
DBP, mean (SD), mm Hg	77.3 (10.8)	77.7 (11.4)	76.7 (10.4)	76.5 (10.5)	78.1 (11.1)	.70
Cholesterol level, mean (SD), mg/dL	184.5 (39.6)	184.5 (38.4)	184.5 (39.5)	180.9 (37.9)	186.3 (42.3)	.89
Creatinine level, mean (SD), mg/dL	0.93 (0.23)	0.93 (0.24)	0.92 (0.23)	0.93 (0.22)	0.93 (0.21)	.89
Cardiovascular disease, No. (%)^b^	96 (20.6)	23 (16.8)	39 (23.2)	13 (25.5)	21 (19.1)	.42

Abbreviations: DBP, diastolic blood pressure; Hp, haptoglobin; NA, not applicable; SBP, systolic blood pressure.

SI conversion factors: To convert glycated hemoglobin level to proportion of total hemoglobin, multiply by 0.01; to convert cholesterol level to millimoles per liter, multiply by 0.0259; and to convert creatinine level to micromoles per liter, multiply by 88.4.

^a^ Denominators are values in the Overall column.

^b^ Denominators are values in the Baseline row.

### Hp Genotype and Baseline MMSE Scores

In the overall analysis of covariance controlling for the sociodemographic variables, the groups differed in their baseline MMSE scores (Table 2): Hp 1-1 had the lowest MMSE score (mean [SE], 25.68 [0.23]) and Hp 2-1m had the highest MMSE score (mean [SE], 27.15 [0.36]). Post hoc comparisons among pairs of the Hp genotypes (eTable 2 in the Supplement), adjusting for multiple comparisons, demonstrated that those with the Hp 2-1m genotype had better scores than those with the Hp 1-1 genotype (mean [SE] difference, 1.46 [0.30]); *P* = .001) and those with the Hp 2-2 genotype (mean [SE] difference, 1.18 [0.44]; *P* = .04). In model 2, the comparison of Hp 1-1 with Hp 2-1m withstood adjustment for multiple comparisons (mean [SE] difference, 1.39 [0.30]; *P* = .007). All other comparisons of pairs of genotypes showed no differences in the baseline MMSE scores.

**Table 2.** Baseline MMSE Scores by Hp Genotype Using the Least Squares Method^a^

Variable	MMSE Score, Mean (SE)	
	Model 1	Model 2
Hp 1-1	25.68 (0.23)	25.74 (0.23)
Hp 2-1	26.26 (0.21)	26.21 (0.21)
Hp 2-1m	27.15 (0.36)	27.13 (0.37)
Hp 2-2	25.97 (0.25)	26.02 (0.25)
*P* value	.006	.01

Abbreviations: Hp, haptoglobin; Mini-Mental State Examination, MMSE.

^a^ Model 1 controls for age, sex, and education. Model 2 controls for age, sex, education, baseline glycated hemoglobin level, systolic blood pressure, diastolic blood pressure, cholesterol level, creatinine level, history of cardiovascular disease at baseline, and glycemic control study arm.

### Hp Genotype and Longitudinal MMSE Score

Table 3 summarizes model 1 and model 2 for longitudinal decline in MMSE score by Hp genotype. The MMSE score declined over time among those with the Hp 1-1, Hp 2-1, and Hp 2-2 genotypes; in the Hp 2-1m group, there was marginal improvement. Using the least squares method, the 40-month decline was significant for Hp 1-1 (mean [SE], −0.41 [0.19]; *P* = .04) and for Hp 2-2 (mean [SE], −0.68 [0.21]; *P* = .001). However, the overall comparison across the 4 groups did not reach statistical significance for model 1 or the fully adjusted model 2 (Table 3 and eFigure 1 in the Supplement).

**Table 3.** Decline in MMSE Score Over 40 Months by Hp Genotype Using the Least Squares Method^a^

Variable	Model 1		Model 2	
	MMSE Score, Mean (SE)	*P* Value	MMSE Score, Mean (SE)	*P* Value
Hp 1-1	−0.39 (0.19)	.04	−0.41 (0.19)	.04
Hp 2-1	−0.26 (0.17)	.13	−0.22 (0.18)	.21
Hp 2-1m	0.16 (0.30)	.59	0.16 (0.31)	.60
Hp 2-2	−0.67 (0.21)	.001	−0.68 (0.21)	.001
*P* value^b^	NA	.14	NA	.13

Abbreviations: Hp, haptoglobin; Mini-Mental State Examination, MMSE; NA, not applicable.

^a^ Model 1 controls for the baseline MMSE score, age, sex, and education. Model 2 controls for the baseline MMSE score, age, sex, education, baseline glycated hemoglobin level, systolic blood pressure, diastolic blood pressure, cholesterol level, creatinine level, history of cardiovascular disease at baseline, glycemic control study arm, use of blood pressure medication at the visit, and use of oral glycemic control medication at the visit.

^b^ *P* value for comparisons among the 4 genotype groups sliced by visit.

### The Role of Age in the Association of Hp With Cognitive Decline

**Figure 2.**
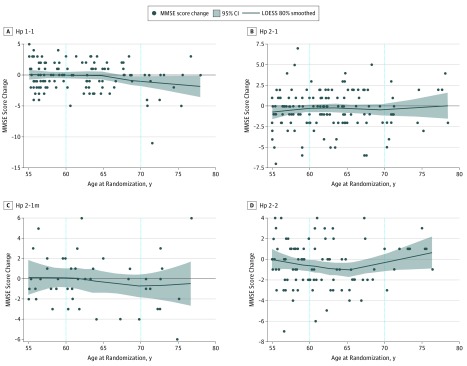
Mini-Mental State Examination (MMSE) Score Change Over 40 Months by Age at Randomization for Each Haptoglobin (Hp) Genotype A-D, The 4 Hp genotypes are shown. LOESS indicates locally weighted smoothing.

As shown in Figure 2, the MMSE score change was subtle for all Hp genotypes in the younger ages (eFigure 2 in the Supplement shows the age distribution of the cohort). The line became significantly steeper for the Hp 1-1 genotype near age 70 years, indicating more decline in older Hp 1-1 carriers. However, in the older age strata, the lines for the other 3 genotypes remained flat. This observation was confirmed by the parametric regression models. The interaction of age with the Hp 1-1 genotype on MMSE score decline estimate per year change was significant (mean [SE], −0.87 [0.37]; *P* = .005), whereas it was not significant for Hp 2-1 (mean [SE], 0.06 [0.37]; *P* = .85), Hp 2-1m (mean [SE], −0.06 [0.51]; *P* = .89), and Hp 2-2 (mean [SE], −0.44 [0.41]; *P* = .29).

### Hp Protein Levels

To explore whether the levels of the protein product of the Hp gene are associated with cognition and cognitive decline and whether they link the Hp genotypes to cognition, we repeated all analyses first using the serum level of Hp protein as the predictor and then including it as a covariate. The Hp protein level was not associated with cognition or cognitive decline, and its inclusion as a covariate in the Hp genotype and cognition models did not alter the results.

## Discussion

In this cohort study of elderly African American adults having long-standing type 2 diabetes and participating in the ACCORD-MIND study, we found suggestive evidence that the Hp 1-1 genotype was associated with poorer global cognitive function and with significant cognitive decline at the 40-month follow-up, adjusting for age, sex, and education as well as when also adjusting for cardiovascular risk factors and the glycemic control study arm. In addition, among Hp 1-1 carriers, older baseline age may modulate the extent of the decline. The Hp protein level was not associated with cognition and did not alter the Hp genotype and cognition associations, suggesting that the structure and function of the Hp genotypes differentially influence cognition. Our study also confirms a 2-fold (approximately 30%) higher prevalence of the Hp 1-1 genotype among African American adults, as shown in another study,^18^ compared with individuals of white race/ethnicity (approximately 14%).^25^ The study by Langlois and Delanghe^18^ focused on African American adults from the Seattle, Washington, area, and the present study broadens to numerous regions in the United States.

Although the data suggest that individuals with different Hp genotypes had some difference in the amount of cognitive decline, the main association of Hp genotype with cognitive decline was not statistically significant. However, there was a significant interaction between age and Hp genotype such that older Hp 1-1 carriers showed significantly greater MMSE score decline over time compared with the other genotypes. Poorer cognitive outcomes in Hp 1-1 genotype carriers may be caused by greater cerebrovascular disease. The Hp 1-1 genotype has been shown to correlate with stroke^14^ and with white matter hyperintensities^13^ in adults with type 1 diabetes. Similarly, in middle-aged patients with hypertension, the Hp 1-1 genotype correlates with the extent of deep white matter damage,^15^ and the frequency of the Hp 1 allele was significantly higher in patients with first symptomatic lacunar stroke due to small vessel disease.^31^ The cluster numbers of endothelial cells, which mitigate cerebral small vessel disease, are lower when Hp 1-1 protein is added to progenitor cell cultures^32^; cluster formation is poorer in patients with lacunar stroke with the Hp 1-1 phenotype.^32^ In contrast, the Hp 2 allele potentiates gene expression of proangiogenic factors in endothelial progenitor cells, potentially improving blood perfusion and recovery from ischemic injury.^33,34^ The association of the Hp 1-1 genotype with cerebrovascular disease and poor cognitive outcomes and the association of the Hp 2-2 genotype with peripheral vascular complications of type 2 diabetes suggest that Hp may serve different functions depending on the neuropathological processes in vascular diseases of different organs. This association merits further investigation because it may have significant clinical implications, suggesting caution against the universal application of therapies targeting vascular disease across all Hp genotypes.

The Hp 2-1m genotype, pertaining solely to individuals of African descent and investigated herein for the first time (to our knowledge) in the context of cognitive aging, was associated with better cognitive function at baseline despite being the group with the oldest age and lowest education. Longitudinally, it was the only group with a suggestion of cognitive improvement over time. Because these results may suggest neuroprotection of this genotype, further investigation is warranted.

### Strengths and Limitations

Strengths of the study include the large sample of African American adults with longitudinal cognitive data and a well-validated diagnosis of type 2 diabetes. The generalizability of the results may be limited because the ACCORD-MIND study sample is derived from a clinical trial and includes participants with severe type 2 diabetes, thus not representing the population of aging individuals with type 2 diabetes. Therefore, the study may have a survivor bias. For example, the Hp 2-2 genotype is associated with greater risk of myocardial infarction.^11^ Because there were no differences herein in the prevalence of myocardial infarction among different genotypes, it is possible that the Hp 2-2 individuals in the ACCORD-MIND study may be those who survived a myocardial infarction. The distribution of the Hp genotypes was similar in those with complete (n = 409) and incomplete data (n = 57) data, suggesting that, in this short-term study, censorship does not depend on Hp genotype. It has recently been shown in older persons of white race/ethnicity with type 2 diabetes that poor glycemic control was strongly associated with hippocampal volume among those with the Hp 1-1 genotype but not among other genotypes.^35^ This outcome suggests that individuals with the Hp 1-1 genotype may be more vulnerable to the deleterious influences of poor glycemic control.

## Conclusions

To our knowledge, this is the first study to investigate the role of the Hp genotype in cognitive function and decline among elderly African American adults with type 2 diabetes, who are at particularly high risk of developing dementia^1,2,3^ and other type 2 diabetes complications.^6,7^ We show that the Hp 1-1 genotype, which is 2-fold (approximately 30%) more prevalent among African American adults than among individuals of white race/ethnicity, was associated with poorer cognitive function and greater cognitive decline than the other Hp genotypes. The Hp gene polymorphism may explain the elevated dementia risk in African American adults. Future research directions should address the neurobiological substrates and mechanisms underlying the associations of Hp and cognition in a more representative sample of older African American adults with type 2 diabetes and with a broader and more sensitive cognitive assessment.

## Notes

Supplement. **eTable 1.** Distribution of Mini Mental State Examination (MMSE) Scores by Hp Genotype **eTable 2.** Comparisons Among Pairs of Hp Genotypes on the Mini Mental State Examination (MMSE) Score Difference (SE) at Baseline **eFigure 1.** Distribution of Age at Baseline **eFigure 2.** Rates of Decline in Mini-Mental State Examination (MMSE) Over 40 Months by Haptoglobin Genotype
