# Supplementary material for: Association of the Haptoglobin Gene Polymorphism With Cognitive Function and Decline in Elderly African American Adults With Type 2 Diabetes: Findings From the Action to Control Cardiovascular Risk in Diabetes–Memory in Diabetes (ACCORD-MIND) Study
Source: JAMA Netw Open. 2018 Nov 9;1(7):e184458. doi: 10.1001/jamanetworkopen.2018.4458 (PMC6324406; doi:10.1001/jamanetworkopen.2018.4458)
Supplement: Supplement. — eTable 1. Distribution of Mini Mental State Examination (MMSE) Scores by Hp Genotype eTable 2. Comparisons Among Pairs of Hp Genotypes on the Mini Mental State Examination (MMSE) Score Difference (SE) at Baseline eFigure 1. Distribution of Age at Baseline eFigure 2. Rates of Decline in Mini-Mental State Examination (MMSE) Over 40 Months by Haptoglobin Genotype [file jamanetwopen-1-e184458-s001.pdf]

Supplementary Online Content

Beeri MS, Lin HM, Sano M, et al. Association of the haptoglobin gene polymorphism with cognitive function and decline in elderly African American adults with type 2 diabetes: findings from the Action to Control Cardiovascular Risk in Diabetes–Memory in Diabetes (ACCORD-MIND) study. *JAMA Netw Open*. 2018;1(7):e184458. doi:10.1001/jamanetworkopen.2018.4458

eTable 1. Distribution of Mini Mental State Examination (MMSE) Scores by Hp Genotype

eTable 2. Comparisons Among Pairs of Hp Genotypes on the Mini Mental State Examination (MMSE) Score Difference (SE) at Baseline

eFigure 1. Distribution of Age at Baseline

eFigure 2. Rates of Decline in Mini-Mental State Examination (MMSE) Over 40 Months by Haptoglobin Genotype

This supplementary material has been provided by the authors to give readers additional information about their work.

**eTable 1.** Distribution of Mini Mental State Examination (MMSE) Scores by Hp Genotype								
**Hp type**	**N Obs**	**Mean**	**Std Dev**	**Minimum**	**Lower Quartile**	**Median**	**Upper Quartile**	**Maximum**
Hp 1-1	137	25.99	2.59	20.00	24.00	26.00	28.00	30.00
Hp 2-1	168	26.23	3.01	17.00	24.00	27.00	29.00	30.00
Hp 2-1m	51	27.12	2.45	22.00	26.00	27.00	29.00	30.00
Hp 2-2	110	26.10	3.04	18.00	25.00	27.00	28.00	30.00

© 2018 Beeri MS et al. *JAMA Network Open*.

eTable 2. Comparisons Among Pairs of Hp Genotypes on the Mini Mental State Examination (MMSE) Score Difference (SE) at Baseline

	**Model 1**				**Model 2**			
**HP type comparison**	**Difference**	**SE**	**Raw p- value**	**Adj p-value***	**Difference**	**SE**	**Raw p- value**	**Adj p-value***
**Hp 1-1 vs. Hp 2-1**	-0.58	0.30	0.05	0.21	-0.47	0.30	0.12	0.40
**Hp 1-1 vs. Hp 2-1m**	-1.46	0.42	<.001	0.003	-1.39	0.43	0.001	0.007
**Hp 1-1 vs. Hp 2-2**	-0.28	0.33	0.39	0.82	-0.28	0.33	0.40	0.83
**Hp 2-1 vs. Hp 2-1m**	-0.88	0.41	0.03	0.14	-0.92	0.42	0.03	0.12
**Hp 2-1 vs. Hp 2-2**	0.29	0.32	0.35	0.79	0.19	0.32	0.56	0.94
**Hp 2-1m vs. Hp 2-2**	1.18	0.44	0.008	0.04	1.11	0.45	0.013	0.06

Adj p-value*: adjusted p-value for multiple comparison using Tukey-Kramer method

© 2018 Beeri MS et al. *JAMA Network Open*.

eFigure 1. Distribution of Age at Baseline.

The median age in the AA participants of the ACCORD-MIND was 61.4; the upper quartile of age averaged 65.3.

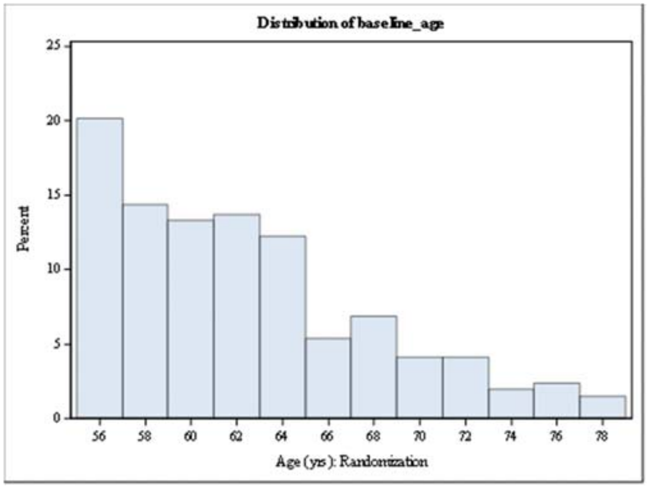

© 2018 Beeri MS et al. *JAMA Network Open*.

eFigure 2. Rates of Decline in Mini-Mental State Examination Over 40 Months by Haptoglobin Genotype (bars=95% confidence intervals)

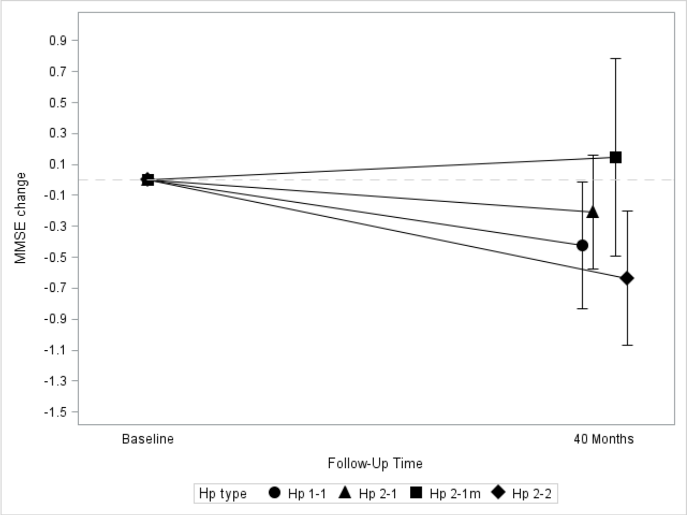

© 2018 Beeri MS et al. *JAMA Network Open*.
